# Observational study on variability between biobanks in the estimation of DNA concentration

**DOI:** 10.1186/1756-0500-2-208

**Published:** 2009-10-13

**Authors:** Jay Brown, Alexander N Donev, Charalampos Aslanidis, Pippa Bracegirdle, Katherine P Dixon, Manuela Foedinger, Rhian Gwilliam, Matthew Hardy, Thomas Illig, Xiayi Ke, Dagni Krinka, Camilla Lagerberg, Päivi Laiho, David H Lewis, Wendy McArdle, Simon Patton, Susan M Ring, Gerd Schmitz, Helen Stevens, Gunnel Tybring, H Erich Wichmann, William ER Ollier, Martin A Yuille

**Affiliations:** 1 Centre for Integrated Genomic Medical Research, University of Manchester, Manchester, UK; 2 School of Mathematics, University of Manchester, Manchester, UK; 3 Institute for Clinical Chemistry and Laboratory Medicine, University Hospital Regensburg, Germany; 4 Health Protection Agency Culture Collections, Health Protection Agency, Salisbury, UK; 5 Clinical Institute of Medical and Chemical Laboratory Diagnostics, Medical University Vienna, Vienna, Austria; 6 Sanger Institute, Wellcome Trust Genome Campus, Hinxton, UK; 7 Cambridge Institute of Medical Research, University of Cambridge, Cambridge, UK; 8 Institute of Epidemiology, Helmholtz Centre, Munich, Germany; 9 Department of Social Medicine, University of Bristol, Bristol, UK; 10 Estonian Genome Project, University of Tartu, Tartu, Estonia; 11 Karolinska Institutet BioBank, Karolinska Institute, Stockholm, Sweden; 12 National Public Health Institute, Biomedicum Helsinki, Helsinki, Finland; 13 National Genetics Reference Laboratory, Central Manchester University Hospitals, Manchester, UK

## Abstract

**Background:**

There is little confidence in the consistency of estimation of DNA concentrations when samples move between laboratories. Evidence on this consistency is largely anecdotal. Therefore there is a need first to measure this consistency among different laboratories and then identify and implement remedies. A pilot experiment to test logistics and provide initial data on consistency was therefore conceived.

**Methods:**

DNA aliquots at nominal concentrations between 10 and 300 ng/μl were dispensed into the wells of 96-well plates by one participant - the coordinating centre. Participants estimated the concentration in each well and returned estimates to the coordinating centre.

**Results:**

Considerable overall variability was observed among estimates. There were statistically significant differences between participants' measurements and between fluorescence emission and absorption spectroscopy.

**Conclusion:**

Anecdotal evidence of variability in DNA concentration estimation has been substantiated. Reduction in variability between participants will require the identification of major sources of variation, specification of effective remedies and their implementation.

## Introduction

Few genotyping labs will receive DNA at a stated concentration and not estimate the concentration again. This occurs because there is little confidence in the consistency of estimations between labs. The receiving lab may then require more DNA for concentration estimation than is needed for the assay itself. The process is likely to be repeated each time the same sample is assayed. When many thousands of samples are to be genotyped, they must be aggregated from multiple biobanks and months are spent standardising concentration and quality.

In the report on the Wellcome Trust Case Control Consortium [1] attention was given to DNA sample quality as a cause of data loss. The report's Table Four shows that one in 21 samples genotyped (809 out of 17,000 DNA samples) were excluded from analysis owing to problems of DNA quality or sample labelling (i.e. data quality). The single most substantial cause of exclusion appeared to be the failure of a DNA sample to attain a single nucleotide polymorphism call rate of >97%. This failure may arise from impurities in the sample, its lack of homogeneity or from inconsistency and or inaccuracy in DNA concentration estimation. Thus, even in well-curated series, time, effort and money may be wasted and an essentially non-renewable resource is depleted.

Medical genomics research requires increasing attention to consistent high quality production and management of both the samples and associated data that are to be the subject of experimental analysis. This attention is necessary because of the need to share resources. Resource sharing involves the aggregation of samples and data from multiple biobanks and, along with improvements in phenotyping [2] is widely recognised as essential for the next generation of genetic epidemiology investigations [3].

Public Population Project in Genomics (P3G) [4] is an organisation of researchers dedicated to fostering collaboration and, thus, resource-sharing, in the field of population genomics. Sharing resources includes aggregation of samples from numerous sources. P3G reasoned that a good starting point to address concerns about sample quality in general [5] was through a focus on DNA. The first issue here is to provide data to support anecdotal evidence that DNA concentration estimations by different laboratories and biobanks are inconsistent. P3G approved [6] a study proposal on this issue from the UK DNA Banking Network [7]. The study proposal planned an observational study undertaken by biobanks that are members of P3G or members of the Biobanking and BioMolecular Resources Infrastructure Preparatory Phase (BBMRI) [8] - the pan-European biobanking [9].

The aim of the pilot study described here is to test the logistics for a larger scale study and to provide some initial data on consistency between biobanks. This study seeks to discover to what extent different laboratories and biobanks obtain different estimates of the concentration of the same DNA solution. No constraint is placed on the technology or instrument used. The results demonstrate substantial variability between participants, instruments and technologies.

An observational study among forensic laboratories of DNA concentration estimation methods and results has been described [10]. Its aims were broader than the study described here, examining the effects of DNA concentration on downstream processes and DNA stability. As far as DNA concentration estimation is concerned, the authors focussed solely on whether a method was quantitative.

## Methods

### DNA preparation and aliquotting

A DNA solution in TE (10 mM Tris, 1 mM EDTA pH 7.5, Invitrogen #T11493) was prepared from three human cell lines at a nominal concentration of 400 ng/μl by the European Collection of Cell Cultures (Salisbury, UK), consistent with appropriate ethical use. The solution was stored (4°C) at the coordinating centre (CIGMR, Manchester). Agarose gel electrophoresis followed by ethidium bromide staining did not detect degradation. The solution was mixed thoroughly in its tube using a Labinco L46 Vortexer for 2 mins at speed setting 10. Volumes were removed manually from the tube into 50 ml tubes (Greiner #227261), diluted with TE to give nominal concentrations of 10, 40, 20, 50, 100, 150, 75, 300 ng/μl (nominal).

For each of the DNA dilutions, either volumes of 20 μl or 40 μl were dispensed into four columns of a 96-well polypropylene plates (ABgene # AB-1058) using a Tecan Freedom 200 liquid handler (Tecan, Switzerland). The plates were shipped on dry ice to each participant (identified here by a number). They were asked to measure DNA concentration in all wells; to use their standard operating procedures for DNA concentration estimation; to return data within 28 days.

Instruments used were as follows: (1) NanoDrop ND-1000; (2) Molecular Devices SpectraMaxPlus384; (3) Thermo Fluoroskan Ascent FL; (4) Tecan GENios; (5) Pharmacia Photometer GeneQuant RNA/DNA Calculator; (6) BMG Labtech FLUOstar Galaxy; (7) Molecular Devices SpectraMax Gemini XPS; (8) Eppendorf Biophotometer. These instruments relied on one of two technologies: absorption at 260 nm and fluorescence excitation (at 485 nm) and emission (detected at 538, 535 or 520 nm). Protocols varied in the number of repeat estimations from four repeats (one participant) to three (six participants) and below.

### Statistical methods

Statistical analysis of the data was carried out using the SAS JMP package [11]. A mixed effect model was fitted to the data, specifying the method of taking measurements and the nominal concentrations as well as their interaction as discrete fixed effects, while the laboratory as random and nested within the methods effect. The properties and the usefulness of mixed effect models have been comprehensively reviewed [12].

## Results and discussion

It was considered advantageous that this study should include academic as well as commercial participants since harmonisation is necessary if genotyping bottlenecks are to be minimised. Recruitment of participants posed little problem. This was probably due to endorsement of the study by P3G and de-identification of participants.

DNA was extracted, diluted and despatched as described. Polypropylene plates containing DNA solutions at nominal concentrations known only to the coordinating centre were despatched to 15 participants. Plates were not despatched to two potential participants because of administrative difficulties with carriers. Improved communication among participants should eliminate this difficulty.

Participants were asked to use their standard operating procedures to estimate DNA concentration and to return data within 28 days. This was achieved in many cases. Improved communications within participants' labs should serve to expedite data return.

Data sets were returned by 13 participants. A total of 2514 observations were reported: 1118 measurements on DNA solutions as provided and 1396 measurements on DNA solutions diluted by participants.

Data were analysed to identify the impact of using different methods in different laboratories for estimating DNA concentrations. The variance of the difference between the measured concentrations and the nominal concentration was seen to increase with the nominal concentration for all methods and for all laboratories. However, the variance of the ratio



was homogeneous. The results of the study were therefore summarised with respect to *R*. When *R *= 1, the measurements confirm precisely the nominal concentrations. There is no expectation that R shall be unity since the measurement of the original DNA stock concentration is not absolute.

Data were analysed for all concentration measurements. A mixed effect model was fitted to the data, specifying the method of taking measurements and the nominal concentrations as well as their interaction as discrete fixed effects, while the laboratory were taken as random and nested within the methods effect. Considering the data as a whole, and based on 1118 observations (compared with 1280 expected observations), the data indicate from the summary of fit, from the analysis of variance and from analysis of lack of fit (Table 1) that there is considerable overall variability. This makes it difficult to draw other conclusions firmly.

**Table 1:** Summary of fit, Analysis

**Summary of fit**				
RSquare	0.591098			
RSquare Adj	0.583644			
Root Mean Square Error	0.26306			
Mean of Response	1.36344			
Observations (or Sum Wgts)	1118			

**Analysis of Variance**				

*Source*	*DF*	*Sum of Squares*	*Mean Square*	*F Ratio*
Model	20	109.73814	5.48691	79.2899
Error	1097	75.91307	0.06920	**Prob > F**
C. Total	1117	185.65122		<.0001

**Lack of Fit**				

*Source*	*DF*	*Sum of Squares*	*Mean Square*	*F Ratio*
Lack of Fit	91	49.191066	0.540561	20.3504
Pure Error	1006	26.722007	0.026563	**Prob > F**
Total Error	1097	75.913072		<.0001
				**Max RSq**
				0.8561

**Figure 1:**
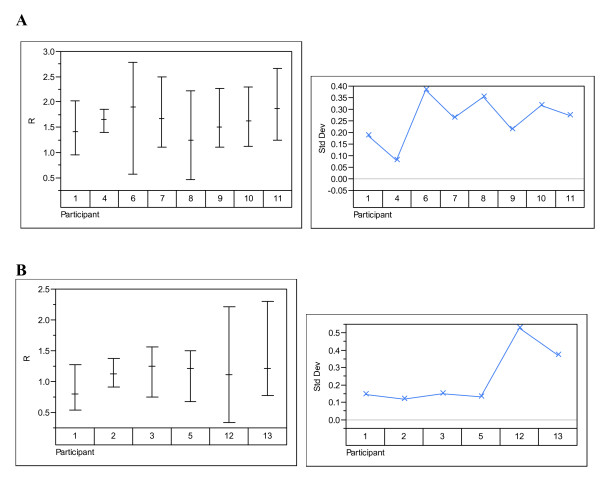
**Variability charts by technology and participant**. Variability charts for R and the standard deviation for each participant using (**A**) absorption spectroscopy and (**B**) fluorescence emission spectroscopy.

**Figure 2:**
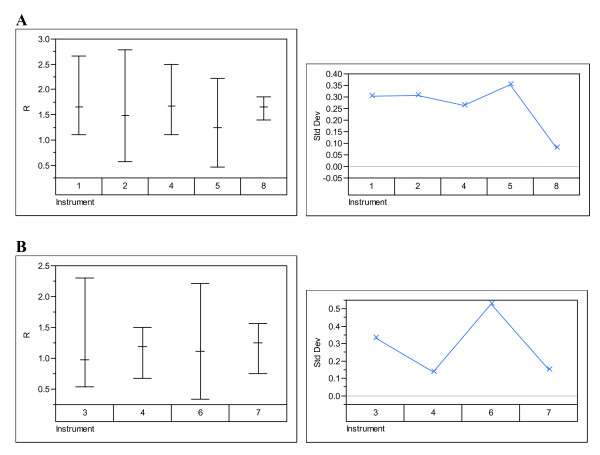
**Variability charts by technology and instrument**. Variability charts for R and the standard deviation for each instrument using (**A**) absorption spectroscopy and (**B**) fluorescence emission spectroscopy. The instrument number identifies one of the instruments listed under Materials and Methods.

**Figure 3:**
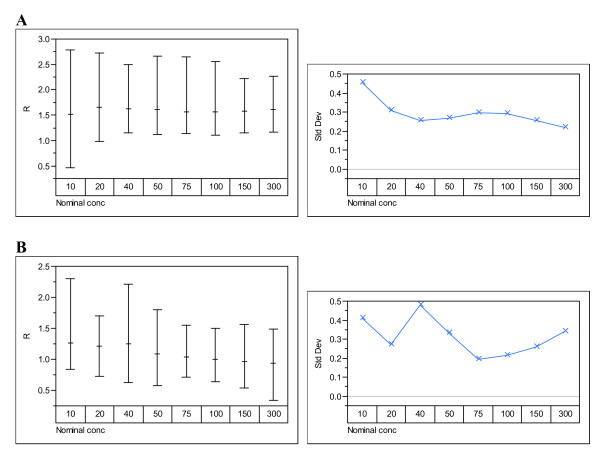
**Variability charts by nominal technology and concentration**. Variability charts for R and the standard deviation at each nominal concentration of DNA provided to participants using (**A**) absorption spectroscopy and (**B**) fluorescence emission spectroscopy.

**Figure 4:**
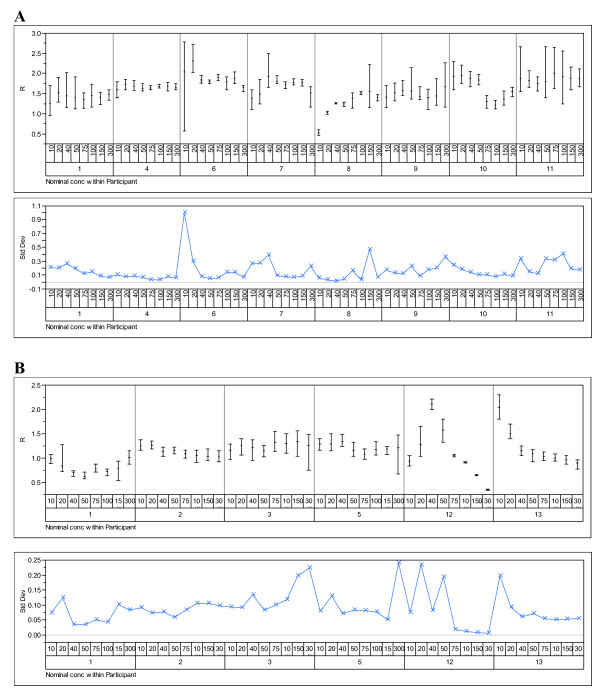
**Variability charts by technology, participant and nominal concentration**. Variability charts for R and the standard deviation for each participant at each nominal concentration of DNA provided using (**A**) absorption spectroscopy and (**B**) fluorescence emission spectroscopy. The instrument number identifies one of the instruments listed under Materials and Methods.

The data were analysed to examine the effects of participant, technology and instrument. Figures 1, 2, 3, 4 show the variability in R by its mean and its standard deviation for each technology by participants (Figure 1), by instrument (Figure 2), by nominal concentration (Figure 3) and by participant and nominal concentration (Figure 4). There was strong evidence that there is a statistically significant difference between technologies: i.e. fluorescence emission produces results showing less variability than produced by absorption spectroscopy. Note that this does not necessarily mean that the former technology is intrinsically less variable than the latter and that therefore the latter technology should be abandoned. It means only that the deployment of the latter technology can generate greater variability than the former. Detailed methods analysis will establish whether it is more practical or efficient or quicker to reduce variability associated with one or the other technology.

There was also strong evidence that there is a statistically significant difference between participants (Figures 1 and 4). However, as all except one participant have used a single method, this difference may be due to the instrument or technology, rather than the participant. Participants 1, 2, 3 and 5 seem to have obtained results with lower variability than the others, although it should be borne in mind that the overall variability subverts this conclusion. Differences between participants may have causes that overlap with the causes of variability from an individual participant. For example, if different operators perform differently within a lab, those differences may be the same for operators in separate labs. Participants that strictly adhere to quality standards such as ISO9001-2000 should show less within-lab variability. However, even if two participants implement strictly a standard such as ISO9001-2000, there may still be substantial between-lab variability owing to lack of identity or lack of precision within one or both of their standard operating procedures or owing to environmental variability.

For each of the nominal concentrations of DNA, variability remained high regardless of technology and participant (Figures 3 and 4). This runs counter to the conventional wisdom that very low or very high DNA concentrations are more difficult to measure accurately. It does not address the question of whether there are the same or different major sources of variability as DNA concentration changes. In this pilot study, no attempt was made to assess variability associated with different DNA molecular weights.

## Conclusion

This is the first reported observational study on DNA concentration estimation among both academic and commercial participants. We have demonstrated the feasibility of an international DNA concentration estimation harmonisation project involving both academic and commercial participants. We provide evidence for significant variation in DNA concentration estimation within and between laboratories. This therefore has confirmed anecdotal evidence for such variation.

This evidence justifies undertaking systematic investigations into the sources of error and the identification, testing, verification and implementation of remedial action that will reduce DNA concentration estimation variability. Such investigations will provide the evidence base for protocol modification. Improvements in the consistency of measurement of DNA are essential for efficient genotyping; for implementing ambitious experimental designs in genetic epidemiology; and for compliance with quality assurance recommendations (e.g. from the Organisation for Economic Cooperation and Development [13]) and requirements (e.g. for continued ISO9001-2000 accreditation).

## Competing interests

The authors declare that they have no competing interests.

## Authors' contributions

CA, PB, KPD, MF, RG, MH, TI, RWJ, DK, CL, PL, DHL, WMcA, SP, SMR, GS, and HS carried out DNA estimations; KPD coordinated the work and carried out DNA estimations; AND and XK performed the statistical analysis; GT, HEW, WERO and MAY conceived the study, participated in its design and helped to draft the manuscript; JB coordinated the work, carried out DNA estimations, participated in the design of the study and helped to draft the manuscript. All authors have read and approved the final manuscript.
